# Unusual apocrine carcinoma with neuroendocrine differentiation: a cutaneous neoplasm may be analogous to neuroendocrine carcinoma with apocrine differentiation of breast

**DOI:** 10.1186/s13000-015-0302-4

**Published:** 2015-06-10

**Authors:** Yang Li, Li-li Chen, Bin Li, Xiao-ying Tian, Zhi Li

**Affiliations:** Department of Pathology, The First Affiliated Hospital, Sun Yat-sen University, 58, Zhongshan Road II, Guangzhou, 510080 China; School of Chinese Medicine, Hong Kong Baptist University, 7, Baptist University Road, Kowloon Tong, Hong Kong, China

**Keywords:** Apocrine carcinoma, Neuroendocrine tumor, Neuroendocrine differentiation, Histogenesis, Differential diagnosis

## Abstract

Cutaneous apocrine carcinoma (AC) is a rare adnexal neoplasm that histologically can mimic breast carcinoma metastatic to the skin or apocrine carcinoma arising in ectopic breast tissue. As extremely rare condition, neuroendocrine differentiation may be observed in AC although its etiology and pathogenesis is still unclear. We report here a case of unusual AC with neuroendocrine differentiation in right labium majus pudenda. A 43-year-old woman presented with a 6-month history of an asymptomatic pea-sized brownish nodule in right labium majus pudenda without enlargement of inguinal lymph nodes and bilateral breast nodules. The mass was totally resected. Microscopically, the tumor was solitary and located in the deep dermis without epidermal connection. Tumor cells were arranged in a micronodular or formed massive solid nests separated by densely fibroblastic stroma. Scattered glandular or rosette-like structures were identified within the tumor nodules. Immunohistochemically, the tumor cells were diffusely positive to CK7, CEA, GCDFP-15, synaptophysin, estrogen and progesterone receptors. Part of tumor cells expressed androgen receptor, but they were negative to CK20, CK5/6, p63 and S-100. Because of its rarity and histogenesis complexity, there exist diagnostic challenges for pathologists to differentiate cutaneous AC with neuroendocrine differentiation from other carcinomas with apocrine or neuroendocrine features. Our case demonstrates that the tumor shares some features with mammary carcinoma and might originate from mammary-like sweat gland in anogenital region. The results suggest that, for the first time, primary cutaneous AC with neuroendocrine differentiation may be analogous to the mammary neuroendocrine carcinoma with apocrine differentiation in histological feature and biological behavior.

**Virtual Slides:** The virtual slide(s) for this article can be found here: http://www.diagnosticpathology.diagnomx.eu/vs/7732276716685708.

## Background

Apocrine carcinoma (AC) is a rare malignant sweat gland neoplasm with apocrine differentiation. It often affects the skin and subcutaneous tissues in axilla and anogenital region of adults without gender bias and racial predilection [1]. In 2006, World Health Organization (WHO) classification of skin tumors have accepted AC as a separate entity, and identified it as a slow growing tumor with a tendency toward a prolonged course, although reliable predictive factors of this tumor have not been established because reports of this tumor are rare and sporadic [2]. In exceedingly rare condition, AC can immunohistochemically and electron microscopically exhibit neuroendocrine differentiation. To the best of our knowledge, only 1 case of AC with neuroendocrine differentiation has been reported in the literature [3]. However, there is no report after this tumor has been established as a distinct entity. Herein, we present an unusual AC with neuroendocrine differentiation occurring in labium majus pudenda of a middle-aged female patient. The clinical and histological features of this tumor, as well as differential diagnosis are discussed.

## Case presentation

### Clinical manifestation and management

A 43-year-old woman presented with an asymptomatic pea-sized brownish nodule in right labium majus pudenda for 6 months. She had no remarkable medical or family history. Clinical examination revealed a dome-shaped elastic nodule, measuring 0.5 cm in diameter in right labium majus pudenda. No cutaneous ulceration was found, and no lymph node was palpable in the bilateral inguinal areas. There was no mass lesion was palpated in the bilateral breast. Routine laboratory test results provided no positive finding, suggesting no bodily biochemical abnormality. The mass excision was performed under the impression of an epidermal cyst or skin fibroma. Intraoperative finding revealed that the mass had no fibrous capsule and located in the deeper dermis. The mass was found to adhere to the underlying tissue, and the border between the mass and subcutaneous fatty tissue was indistinct. However, the mass was not observed to invade the epidermis. The mass was totally removed.

### Pathological findings

**Fig. 1:**
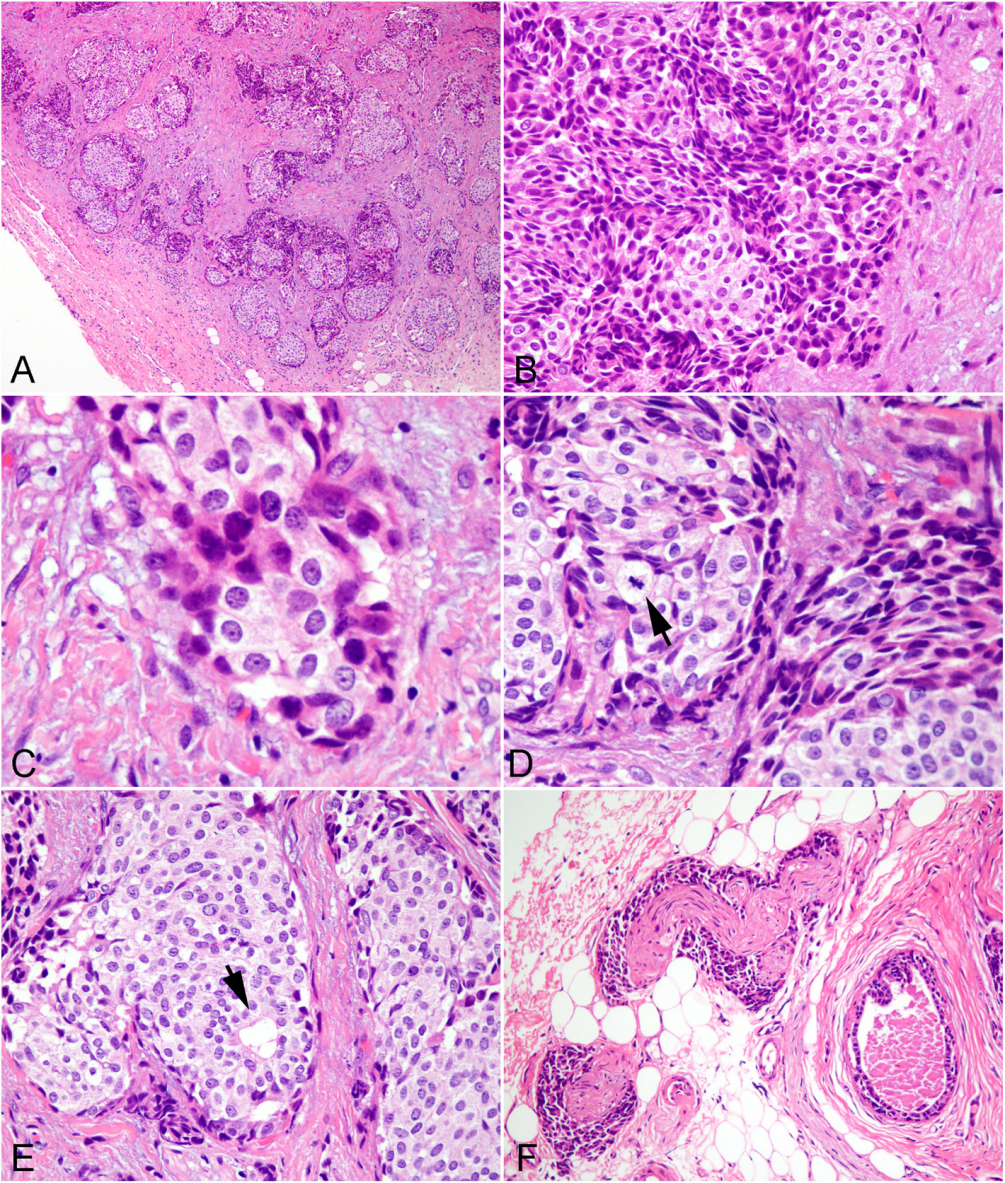
Micrographs of cutaneous mass in postoperative histological examination. **a** A solitary well-circumscribed mass was found in the deep dermis without epidermal connection. The tumor cells formed nodule or solid nests separated by densely fibroblastic stroma. **b** Higher magnification showed the two types of tumor cell mixed in the solid nest. **c** Under the high power field, one type of tumor cell was round to polygonal cells, which had eosinophilic, relatively narrow cytoplasm. The other had abundant pale or foamy cytoplasm. Both two types of tumor cell had enlarged nuclei with distinct nucleoli. **d** The tumor cells showed mild to moderate atypia with scattered mitotic figures (black arrow). **e** There were scattered glandular or rosette-like structures identified within the tumor nodules (black arrow). **f** The tumor cells were observed to spread into the subcutaneous fatty tissue with neural invasion. **a**, H&E staining with original magnification × 100; **b**-**f**, H&E staining with original magnification × 400)

**Fig. 2:**
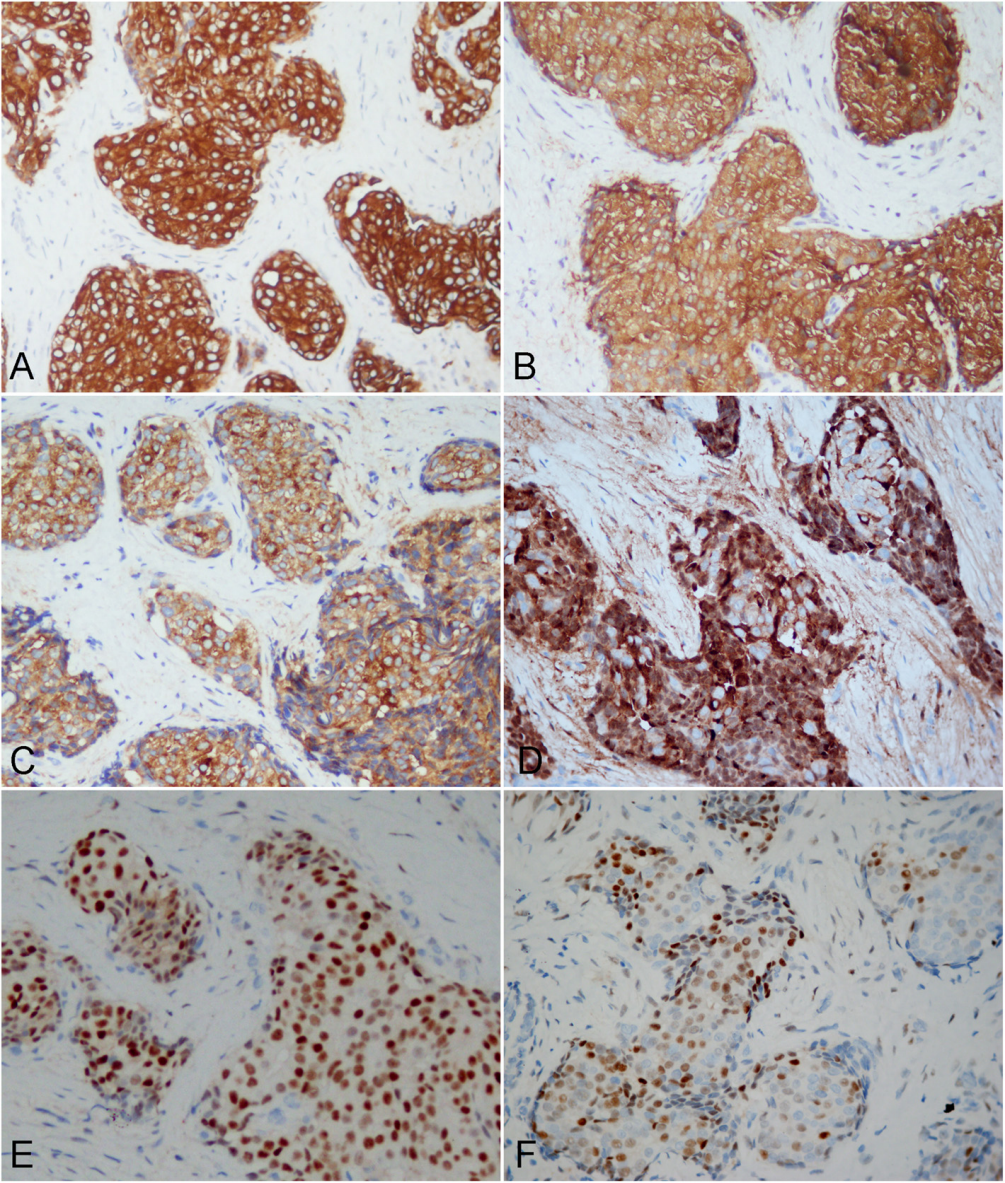
Immunohistochemical features of cutaneous mass. Immunohistochemically, the tumor cells were diffusely immuno-positive to CK7 **a**, CEA **b**, Synaptophysin **c**, GCDFP-15 **d**, and estrogen receptor **e**. **f** About 50 % of tumor cells were detected to be positive to androgen receptor (AR). (**a**-**f**, immunohistochemical staining with original magnification × 400)

The surgical specimen was received and routinely fixed in 10 % neutral buffered formalin after tumor resection. Four micrometer thick sections were cut and stained with H&E. Microscopic examination revealed the tumor was solitary and located in the deep dermis without epidermal connection. The mass had an infiltrating border, and tumor cells were observed to spread into the subcutaneous fatty tissue with neural invasion. The tumor was composed of two types of cells. One was round to polygonal cells, which had eosinophilic, relatively narrow cytoplasm. The other had abundant pale or foamy cytoplasm. Both two types of tumor cell had enlarged nuclei with distinct nucleoli. These tumor cells were arranged in a micronodular or formed massive solid nests separated by densely fibroblastic stroma. However, scattered glandular or rosette-like structures representing ductal lumina were identified within the tumor nodules. There was no bluish mucinous material observed in the duct lumina. Both tumor cells showed mild to moderate atypia with low mitotic activity (1/10 high power fields). Neither tumor necrosis nor hemorrhage was noted in the tumor (Fig. 1). Immunohistochemically, the tumor cells were diffusely immuno-positive to pan-cytokeratin (Pan-CK), CK7, epithelial membrane antigen (EMA), carcinoembryonic antigen (CEA), gross cystic disease fluid protein (GCDFP)-15. More than 80 % of the tumor cells were also reactive to estrogen receptors (ER) and progesterone receptors (PR), synaptophysin and chromogranin A. About 50 % of tumor cells were detected to be positive to androgen receptors (AR), but both two types of tumor cell did not express CK20, mammaglobin and myoepithelial cells markers, such as CK5/6, S-100 protein and p63 (Fig. 2). Alcian blue staining showed there was no intra-cytoplasmic or intra-glandular mucin production in the tumor.

On the basis of gross, histopathological features and immunohistochemical phenotypes, its dermal location, the presence of infiltrating growth pattern and neural invasion, a diagnosis of cutaneous apocrine carcinoma with neuroendocrine differentiation was made. Since there was a possibility of skin metastatic carcinoma from apocrine mammary carcinoma, the patient was referred to a whole body positron emission tomography (PET)/CT study to search for the potentially breast tumor, but no abnormality was found. Because the margin of the tumor was ill-defined, the second excision with a wide margin (1 mm to the excision margin) was performed after diagnosis. The section margin was free. The postoperative phase was uneventful, and no additional treatments were undertaken. The patient was on regular follow-up for 12 months after discharging from hospital. There was no sign of tumor recurrence and lymph node enlargement.

## Discussion

Cutaneous apocrine carcinoma (AC) is a rare adnexal neoplasm that histologically can mimic breast carcinoma metastatic to the skin or apocrine carcinoma arising in ectopic breast tissue. Most cutaneous AC arises in the axilla or anogenital region. But rare locations, such as the scalp, face, and extremities have also been described [4, 5]. It can present with a wide range of clinical modalities and frequently occur in the adults with an average age of 57.9 years [6]. In many cases, the lesions have been standing for more than 10 years, and even up to 30 years before diagnosis [2]. The etiology of AC is unknown. The fact that all patients were over 25 years suggests that full maturity of the apocrine gland is a prerequisite. Although AC is thought to arise from apocrine gland, an interesting alternative origin is the newly described mammary-like sweat gland of the anogenital region [7]. In the present case, the tumor locates in the deep dermis of anogenital region, whether or not it derives from mammary-like sweat gland should be clarified further.

Despite its enigmatic histogenesis, AC with neuroendocrine differentiation is extremely rare. To the best of our knowledge, only 1 case have been reported in the literature, and there is no report after this tumor is established as a distinct entity in 2006 [3]. In that case, a solitary tumor was found in the pubic area. The tumor was composed of polygonal atypical cells in a cord- or ribbon-like fashion. Like our presenting case, the tumor cells stained positive to CEA, GCDFP-15, CK7, chromogranin A, progesterone and androgen receptors. Membrane-bound dense core granules, representing neurosecretory granules, were ultrastructually found in some of the cells. The author suggests that AC with neuroendocrine differentiation shares several features with mammary carcinoma [3].

At birth, the apocrine sweat glands are located primarily on the axilla and in the anogenital region. However, modified apocrine glands exist in the eyelid (Moll’s gland), in the ear canal (ceruminous glands), and in the breast as mammary glands [8]. Several tumors with identical morphologies can occur in these organs. For example, endocrine mucin-producing sweat gland carcinoma is analogous to the mammary solid-papillary carcinoma histopathologically and immunohistochemically [9, 10]. Whether or not primary cutaneous AC with neuroendocrine differentiation has also an analogous lesion in breast? To our knowledge, there are two histotypes of breast cancer exhibit apocrine morphological and immunohistochemical features. They are carcinoma with apocrine differentiation and neuroendocrine carcinoma with apocrine differentiation. Mammary carcinoma with apocrine differentiation is composed of type A and type B cells with typically GCDFP15 and androgen receptor positive, estrogen and progesterone receptors negative [11]. The presence of cells with co-expression of apocrine and neuroendocrine markers has never been described in this specific subtype of breast cancer. In our case, two cell types with distinct morphological features are similar to the type A and B cells identified in the carcinoma with apocrine differentiation. However, we ruled out this diagnosis on the basis of the positive expression to estrogen and progesterone receptors, as well as synchronic neuroendocrine markers expression.

Neuroendocrine carcinoma with apocrine differentiation is a rare variant of breast neuroendocrine carcinoma with “divergent” differentiation [12, 13], although it has not been described in the most recent WHO’s classification of tumor of breast [14]. Few case reports of breast neuroendocrine carcinoma with apocrine differentiation have demonstrated that this tumor is closely associated with strong expression of estrogen, progesterone and androgen receptors [12, 13, 15]. However, only approximately 60 % cases of primary cutaneous AC showed ER and PR positive in tumor cells. [16]. In present case, we found some analogous features between AC with neuroendocrine differentiation and mammary neuroendocrine carcinoma with apocrine differentiation. Morphologically, both tumors are composed of multiple circumscribed cellular and solid nodules separated by densely fibroblastic stroma. Each nodule comprises polygonal to ovoid cells with abundant eosinophilic granular cytoplasm forming focal rosette-like structures or small ductal lumina. Immunohistochemically, both tumors co-express neuroendocrine and apocrine markers diffusely. More importantly, similar to neuroendocrine carcinoma with apocrine differentiation, AC with neuroendocrine differentiation also expresses estrogen and progesterone receptors. These similar features might imply the correlation between these two distinct tumors. In agreement with previous studies, our result supports the hypothesis of the AC having originated from mammary-like sweat gland of the anogenital region. We presume that primary cutaneous AC with neuroendocrine differentiation, similar to its mammary counterpart, neuroendocrine carcinoma with apocrine differentiation, could present analogous characteristics in histomorphology and biological behavior.

Because of histological resemblance, many authors consider that ACs located in axillary region is indistinguishable from metastatic breast adenocarcinoma [17–19], and that a detailed clinical pathological correlation needs to be established to distinguish between them [2, 20]. As for cutaneous AC with neuroendocrine differentiation, it could be distinguished from metastatic breast carcinoma with apocrine differentiation by the presence of co-expression of apocrine and neuroendocrine markers in tumor cells, and the absence of primary carcinoma in bilateral breast. However, it might be indistinguishable from neuroendocrine carcinoma with apocrine differentiation of ectopic breast tissue in axillary or anogenital region. Since the AC in the anogenital region might arise from mammary-like sweat gland, in fact, we have no idea to confirm if our case is indeed a neuroendocrine carcinoma with apocrine differentiation of ectopic breast tissue in anogenital region even if there is absence of ectopic mammary tissue in the sample. The morphological features and immunohistochemical staining are non-specific for their differentiation. The accurate cliniopathological criteria need to be established further.

There are no standardized protocols for the treatment of AC. Wide local excision with margins between 1 and 2 mm has been recommended. The previously reported case of AC with neuroendocrine differentiation exhibited a poor prognosis, the patient died of regional lymph node metastasis and multiple pulmonary metastasis 22 months after surgical excision [3]. However, several cases of breast neuroendocrine carcinoma indicated that the association with apocrine differentiation seemed to improve long-term survival of patients with neuroendocrine carcinoma [12, 15]. Regardless of its ectopic breast origin, in case cutaneous AC with neuroendocrine differentiation is analogous to the mammary neuroendocrine carcinoma with apocrine differentiation, we postulate a favorable prognosis in our case. Of course, a longer period of follow-up is necessary to be performed to supervise the tumor progression.

## Conclusion

Because of its rarity and histogenesis complexity, there exist diagnostic challenges for pathologists to differentiate cutaneous AC with neuroendocrine differentiation from other carcinomas with apocrine or neuroendocrine features, especially when the tumor occurs in axillary and anogenital region. It is important to be able recognize this tumor in order to avoid potential misdiagnosis and improper management of afflicted patients. This tumor shares some features with mammary carcinoma and might originate from mammary-like sweat gland. Our results suggest that, for the first time, primary cutaneous AC with neuroendocrine differentiation may be analogous to the mammary neuroendocrine carcinoma with apocrine differentiation in histological feature and biological behavior.

## Consent

Written informed consent was obtained from the patient for publication of this case report and any accompanying images. A copy of the written consent is available for review by the Editor-in-Chief of this journal.
